# Ramped pulse shapes are more efficient for cochlear implant stimulation in an animal model

**DOI:** 10.1038/s41598-020-60181-5

**Published:** 2020-02-24

**Authors:** Charlotte Amalie Navntoft, Jeremy Marozeau, Tania Rinaldi Barkat

**Affiliations:** 1 Hearing Systems Group, Department of Health Technology, Technical University of Denmark, Kgs. Lyngby, Denmark; 2 Brain and Sound Lab, Department of Biomedicine, Basel University, Basel, Switzerland

**Keywords:** Neuroscience, Medical research

## Abstract

In all commercial cochlear implant (CI) devices, the electric stimulation is performed with a rectangular pulse that generally has two phases of opposite polarity. To date, developing new stimulation strategies has relied on the efficacy of this shape. Here, we investigate the potential of a novel stimulation paradigm that uses biophysically-inspired electrical ramped pulses. Using electrically-evoked auditory brainstem response (eABR) recordings in mice, we found that less charge, but higher current level amplitude, is needed to evoke responses with ramped shapes that are similar in amplitude to responses obtained with rectangular shapes. The most charge-efficient pulse shape had a rising ramp over both phases, supporting findings from previous *in vitro* studies. This was also true for longer phase durations. Our study presents the first physiological data on CI-stimulation with ramped pulse shapes. By reducing charge consumption ramped pulses have the potential to produce more battery-efficient CIs and may open new perspectives for designing other efficient neural implants in the future.

## Introduction

**Figure 1:**
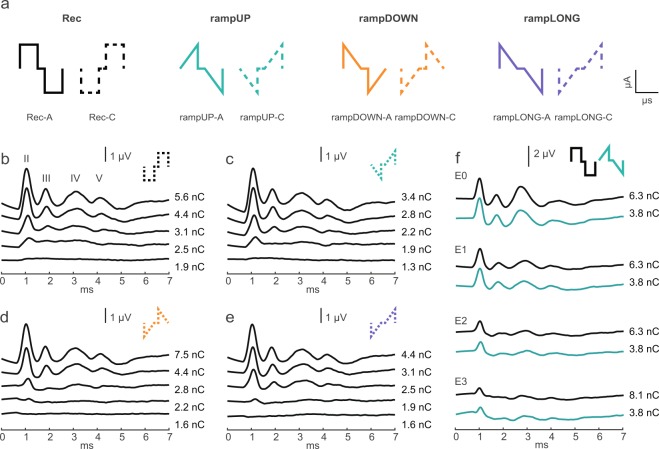
Ramped pulses can evoke eABRs. (**a**) Pulse shapes used. Pulses were charge-balanced and biphasic with 25 μs/phase and a 10 μs interphase gap. Rectangular (Rec) and ramped (Ramp) pulses are shown from left to right. The polarity of the first phase is indicated by -A (anodic) or -C (cathodic), respectively. (**b–f**) Representative eABR response traces to increasing stimulation level on E0 (the most apical electrode) to (**b**) rectangular, (**c**) rampUP, (**d**) rampDOWN, and (**e**) rampLONG, all with cathodic-first polarity (mouse M2). (**f**) Example responses to Rec (black) and rampUP (green) on all four intracochlear electrodes, both with anodic-first polarity (mouse M7). The amount of injected charge used is written at each trace level. Time 0 ms is defined as onset of the stimuli artefact (removed by linear interpolation). eABR waves are denoted by roman numbers.

Cochlear implants (CIs) can provide deaf people with a sense of hearing by directly stimulating the auditory nerve. Although CI users can perceive speech in quiet settings fairly well, they face challenges when it comes to more complex sound environments, such as understanding a friend speaking in a noisy restaurant or perception of music. These limitations are in particular caused by the spread of current induced by each electrode and by the poor efficiency between the electrical pulse and auditory-nerve responses. As a consequence, each intracochlear electrode activates an inappropriately broad range of auditory nerve fibers and the number of independent channels that a CI user can perceive is thus reduced from a maximum of 22 physical electrodes to about 6–8^1^. In all commercial CI devices, the electrical pulse has a rectangular shape and usually consists of two phases of opposite polarity to ensure charge-balanced stimulation. This configuration renders them safe for long-term usage by CI users. However, it is not the most efficient way to stimulate neurons because the charge injected is immediately counteracted by the following phase^2,3^. In fact, recent evidence gathered by using patch-clamp recordings *in vitro*^4^ and supported by modeling work^5,6^ suggests that the use of an alternative, non-rectangular pulse shape with a ramped slope (Fig. 1a) might be more efficient. In this study, we explore this idea further and test biophysically-inspired electrical pulse shapes *in vivo*.

The arguments for using a ramped shape pulse stimulation strategy rely on two features of the spiral ganglion neurons (SGN) and on diffusion in an aqueous media. First, ramped pulses reportedly match ion channel dynamics of SGN better^4^. SGNs, among other neurons in the auditory system, express low-threshold potassium channels (KLT)^7^. These ion channels are sensitive to the rate of change of a synaptic input current and regulate firing patterns, in particular adaptation to high stimulation rate, by extending the refractory period and a sharp timing of the action potential^7–11^. For example, in patch clamp recordings on cultured SGNs, Ballestro *et al*. showed that the stimulus efficiency needed to evoke action potentials increased as the stimulus slope became steeper^4^. Also, inclusion of KLT channels in a Hodgkin & Huxley-based model of the auditory nerve could better predict adaptation, accommodation (subthreshold adaptation) and absolute refractory periods^9,10^. Including KLT channels also provided better predictions of threshold and spike times for both anodic (positive) and cathodic (negative) pulse shapes in recordings of single auditory nerve fibers^12^. Second, the current of ramped pulses have a more beneficial diffusion pattern in the perilymph fluid in the cochlear^4^. With the standard rectangular pulse shape, the current level amplitude decreases with distance from the stimulated electrode and the rectangular shape is maintained. As a consequence, many neurons are activated. However, if the stimulation is performed by using a pulse shape with a slope, which decreases both in current level amplitude and steepness with distance from the stimulated electrode, then fewer neurons are activated further away from the stimulated electrode because of a shallower slope (cf. Figure 3 in Ballestro *et al*.^4^).

Using ramped pulses for CI stimulation has several potential benefits, including less channel interaction, larger perceptual dynamic range, and reduced battery consumption^4^. Regarding channel interaction, if ramped pulses excite a more focused population of auditory nerve fibers, it would eventually provide more independent channels for information to be transmitted through. Even though previous studies have shown that speech perception does not improve beyond 4 to 10 channels of information^13,14^, two recent studies have suggested that speech performance in noise can improve with more than 8^15^ or 12 channels^16^. Concerning perceptual dynamic range, CI users have a limited electrical dynamic range (often < 10 dB), compared to the 120-dB acoustic dynamic range observed in listeners with normal hearing. This reduced range is partially because electric stimulation generates highly synchronous activity across the excited neural population. That contrasts with acoustic stimulation which produces much less across-fiber synchrony and much more within-fiber jitter due to the stochastic properties of the hair cell-SGN synapse. Ramped pulses could potentially reintroduce some degree of stochasticity by a variable expression of KLT channels, and thus graded sensitivity, to ramped pulses across SGNs. This would result in a more gradual recruitment of auditory nerve fibers with increasing current level, thereby mimicking acoustic hearing, and increasing the dynamic range. Finally, with regard to battery consumption, two studies have reported that ramped pulses are more power-efficient than rectangular pulses in models of the auditory nerve^5,17^. Importantly, Yip *et al*. recently demonstrated the efficiency of novel non-rectangular pulse shapes in human CI experiments^5^. In a loudness perception task, they found that an exponential waveform (not linear as used in Ballestro *et al*.^4^) with five discrete current steps produced up to 26% energy savings within a comfortable range of hearing compared to a rectangular waveform^5^. Lower power consumption reduces battery usage, which means that batteries in the sound processor behind the ear can be smaller and less visible, or that the CI users may need to change them less frequently. Also, in the long run, more efficient batteries might also motivate the development of the invisible, fully-implantable CI devices. In summary, previous studies suggest many advantages to using ramped instead of rectangular pulses for CI stimulation but it remains yet to be confirmed with physiological data.

In this context, the aims of the present study are (1) to characterize responses to ramped pulse shapes using animal eABR recordings, and (2) to test the hypothesis that ramped pulses are more charge-efficient than rectangular pulses *in vivo*. The outcome has the potential to produce more efficient CIs in terms of battery use and to guide future pulse shape design so that speech perception in CI users may improve.

## Results

### Ramped shapes can evoke eABRs

Auditory brainstem response (ABR) is a compound potential evoked in response to auditory stimuli. It is characterized by a set of waves that represent different stations along the auditory pathway. To first test if ramped pulses could generate an electrically-evoked ABR (eABR), we presented trains of both rectangular and ramped pulses to the most apical electrode (E0) and measured eABRs in the first group of mice (n = 12). The four pulse shapes produced highly similar eABR wave patterns, as shown from one mouse (Fig. 1b–e). Stimulation at more basal electrodes with the same amount of charge also evoked an eABR response but with smaller wave amplitudes, as demonstrated with Rec and rampUP pulses (Fig. 1f). The smaller eABR amplitude observed with more basal electrode-stimulation is likely due to the slightly longer distance from the basal electrodes to the modulus relative to the apical electrodes, as was recently indicated in a x-ray image of a CI-implanted mouse cochlea^18^. This is also supported by a study in guinea pigs which showed that electrode position within scala tympani affects eABR threshold^19^.

In the following, only wave II was quantified because the peak and trough were reliably evoked by all four pulse shapes. For the same reason, only stimulation of the most apical electrode was considered.

### Ramped shapes require less charge than rectangular shapes to evoke an eABR

**Figure 2:**
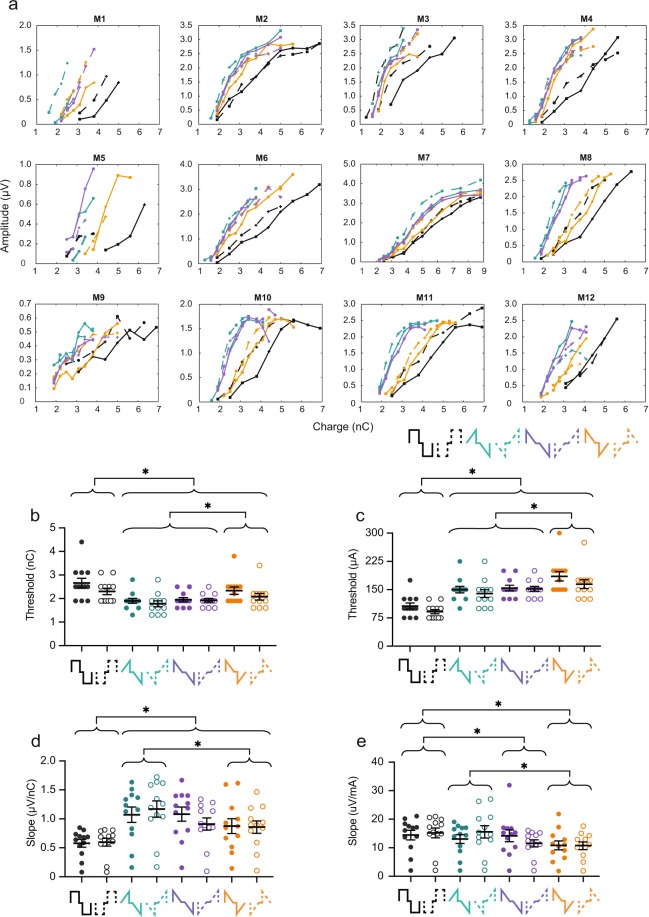
Ramped pulse shapes produce a lower threshold and steeper growth function of eABR wave II compared to rectangular shapes in terms of injected charge. (**a**) Individual amplitude growth functions of wave II as a function of charge injected (Mouse (M)1–12). Pulse shapes are indicated by color and shown in the legend (bottom right). Solid line, anodic-first; dashed, cathodic-first. (**b**) Thresholds of wave II in charge. Pulse shapes are shown below the x-axis. Each circle is data from one mouse. Closed circles, anodic-first pulses; open circles, cathodic-first pulses. (**c**) Thresholds of wave II in amplitude current level. (**d**) Slopes of the growth function of wave II in charge (μV/nC). (**e**) Slopes of the growth function of wave II in amplitude current level (μV/μA). Asterisks denote significant effect of the factor pulse shape in post-hoc test. *p ≤ 0.05. n = 12. Error bars are SEM.

Earlier studies have argued that ramped shapes require less charge to evoke responses similar in amplitude to those from rectangular pulses^4,5,17^. To test this hypothesis *in vivo*, we first plotted the amplitude of wave II as a function of charge injected per phase for all mice (Fig. 2a). The dynamic range displayed was from threshold to the charge level before a facial nerve response was evoked or until the response reached saturation. Different types of growth functions are observed. Mice M1 and M5 display linear curves with a low measurable dynamic range. The curves for mice M3, M4, M6, M8, M9 and M12 are also linear, but with a larger dynamic range. The curves for mice M2, M7, M10 and M11 are linear at low to medium charge levels, and saturated at higher charge levels. These differences could be due to small variations in cochlear anatomy, facial nerve location, position of the electrode inside the scala tympani, path of the current flow from stimulation contact to ground (including impedance), and placement of recording electrodes. Nonetheless, the general pattern across mice is that of a leftward shift of the ramped shapes relative to the rectangular shape. That implies there are lower charge levels for similar responses with ramped shapes. In most animals, the order of efficiency was rampUP (green), rampLONG (purple), rampDOWN (orange), Rec (black) (Fig. 2a). Cathodic-first pulses also required less charge to evoke responses of similar amplitude relative to anodic-first pulses (Fig. 2a).

Next, we compared wave II thresholds across the four pulse shapes (Fig. 2b). A mixed model with pulse shape (Rec, rampUP, rampDOWN, rampLONG) and polarity (anodic or cathodic-first) as fixed factors and mouse as a random factor showed a highly significant effect of pulse shape (n = 12, P < 0.0001) and of polarity (P = 0.0011) on the threshold. The pulse shape*polarity interaction was not significant (P = 0.0853). Post hoc comparison (Tukey HSD) showed that the thresholds for the three ramped shapes were all significantly lower than for Rec in terms of charge. Interestingly, the threshold of rampUP and rampLONG were also significantly lower than that of rampDOWN. If SGN are sensitive to a linear rising current (injected current that increases in magnitude with a constant, rising slope) but not to a linear declining current (injected current that decreases in magnitude with a constant, declining slope) ramp^4^, then we would have expected rampUP, with a constant rising slope in both phases, to produce the lowest threshold. The second phases of both rampUP and rampLONG have a constant rising slope. One possible explanation for the lower threshold we observed with both rampUP and rampLONG could be that less charge is needed to evoke a response when the second phase has a constant rising slope, independent of the direction of the slope of the first phase. It is known that increasing interphase gaps reduces behavioral and physiological thresholds^2,20–22^. Another, not mutually exclusive, explanation could therefore be that the linear increase of current in the second phase is equivalent to a longer interphase gap, which would also result in a lower threshold. This hypothesis is tested further down. In addition, cathodic-first pulses had lower thresholds than those of anodic-first pulses, which is in line with polarity effects found in previous studies on animals^2^ and in computational models^23^. The threshold ratio between anodic- and cathodic-first pulses averaged across pulse shape was 1.10, which means that cathodic-first pulses were 1.10 times more efficient than anodic-first pulses. Thus, our results show that ramped pulses require less charge to evoke a wave II response compared to the rectangular ones, and that the direction of the ramp matters.

The eABR threshold depends on various stimulation parameters, including amount of injected charge, phase duration, interphase gap length, presentation rate, electrode position, amplitude current level, etc. We therefore also compared wave II thresholds in terms of current level amplitudes (Fig. 2c). Similar to the charge thresholds (Fig. 2b), we observed that pulse shape (P < 0.0001) and polarity (P = 0.0001) had significant effect on the current threshold. The pulse shape*polarity interaction was not significant (P = 0.0905). Post hoc comparison (Tukey HSD) showed the opposite of Fig. 2b: current threshold for all three ramped shapes were significantly higher than those for Rec (P < 0.05). The current thresholds of rampUP and rampLONG were still significantly lower than that of rampDOWN (P < 0.05). These results demonstrate that ramped pulses require less charge but higher current level amplitude to evoke a wave II response than rectangular pulses do, and that rampUP and rampLONG are most efficient.

Similar conclusion could be reached for the threshold of eABR wave III and wave IV (Supplementary Fig. 1a,b,e,f, respectively).

Finally, to investigate if individual eABR wave II thresholds were related the hearing status before deafening, eABR thresholds were averaged across polarities to obtain one value for each of the four pulse shapes and plotted against pre-deafening aABR thresholds (Supplementary Fig. 2a,b). However, none of the four relations was significantly correlated.

### Ramped shapes have steeper eABR growth function slopes compared to rectangular shapes

ABR reflects evoked synchronous activity. The growth function of an ABR wave is therefore determined by how many synchronized neurons and at which speed they are recruited. To examine if the wave II growth function slope is dependent on the pulse shape, we obtained the individual slopes produced by each pulse shape. We next fitted a ‘broken-stick’ model (see Methods) to four mice (M2, M7, M10 and M11) because these functions reached saturation, and a linear function to the remaining mice because these functions increased monotonically based on visual inspection. Two mice (M5 and M9) had low slope values across all pulse shapes, but as the thresholds of these mice were within 2 standard deviations from the mean, they were not excluded from the analysis. Pulse shape, polarity, and type of fit (linear or broken-stick) had no significant effect on the goodness of the fit (R-squared values). When leaving out the type of fit, however, the mixed model showed that pulse shape had a highly significant effect on the growth function slope (P < 0.0001) (Fig. 2d). The interaction between pulse shape*polarity was significant too (P = 0.0002), while polarity alone had no significant effect (P = 0.7147). Post hoc analysis (Tukey HSD) specified that the slopes were significantly higher for rampUP, rampDOWN and rampLONG pulses than for Rec, and that the slope of rampUP was significantly higher than that of rampDOWN (P < 0.05). In contrast to the threshold values, the slope of rampLONG was not significantly different from that of rampDOWN.

In terms of charge, our findings suggest that the maximum response can be obtained with less charge (and potentially less battery use) when ramped shapes are used instead of rectangular shapes, and that the direction of the pulse shape ramp matters. A rising ramp (rampUP) is more charge-efficient than a declining ramp (rampDOWN), which supports the hypothesis of benefits from a rising ramp. We also tested the slope values in terms of amplitude current level (μV/mA) (Fig. 2e). Pulse shape was a highly significant factor (P = 0.0001), and post hoc comparison (Tukey HSD) showed that the slope of Rec was significantly higher than that of rampDOWN and that of rampLONG (P < 0.05). It also showed that the slope of rampUP was significantly higher than of rampDOWN (P < 0.05) (Fig. 2f). Rec and rampUP were not significantly different. In terms of current amplitude, our results suggest that Rec and rampUP generate similar growth function rates, and that the direction of the pulse shape ramp again matters because rampUP and rampDOWN were different.

Similar conclusion could be reached for the slope of eABR wave III but not wave IV (Supplementary Fig. 1c,d,g,h, respectively).

To investigate if individual eABR wave II slopes were related to individual differences in aABR growth functions, eABR slopes were plotted against aABR wave II slopes (Supplementary Fig. 2c,d). However, none of the four relations was significantly correlated. These results suggest that individual differences in growth functions cannot account for the variations in threshold and slope values of wave II evoked with CI-stimulation across animals, respectively.

In general, a steeper growth function implies a higher recruitment rate of neurons with increasing current. Possible reasons for this could on one hand be different sites of excitation, larger current spread, stronger synchronization or recruitment of a more uniform neural population. On the other hand, the findings could be attributed to higher efficiency of the input stimuli and better integration of charge thereby improving the electrode-neuron interface.

As a final note, a steeper growth function also implies that maximum response is reached with less charge. The upper limit of electric stimulation was in most mice determined by the presence of a facial nerve response. Facial nerve stimulation is one of the most well-known and most frequent complications in the cochlear implantation procedure^24^. It is therefore of high clinical relevance to try to minimize it. To investigate if ramped pulses produce less facial nerve stimulation, we plotted the facial nerve response as a function of pulse shape (Supplementary Fig. 3). A significantly higher level of current, but lower amount of charge, could be used with ramped pulses before a facial nerve response was evoked compared to when rectangular pulses were used (Tukey HSD) (Supplementary Fig. 3a,b). Thus, stimulation with ramped pulses could be beneficial if the reduction in charge needed to reach the most comfortable level is larger than that needed to excite the facial nerve. Clinical studies are needed to provide further insight into this aspect.

### rampDOWN have shorter wave II latency than Rec

**Figure 3:**
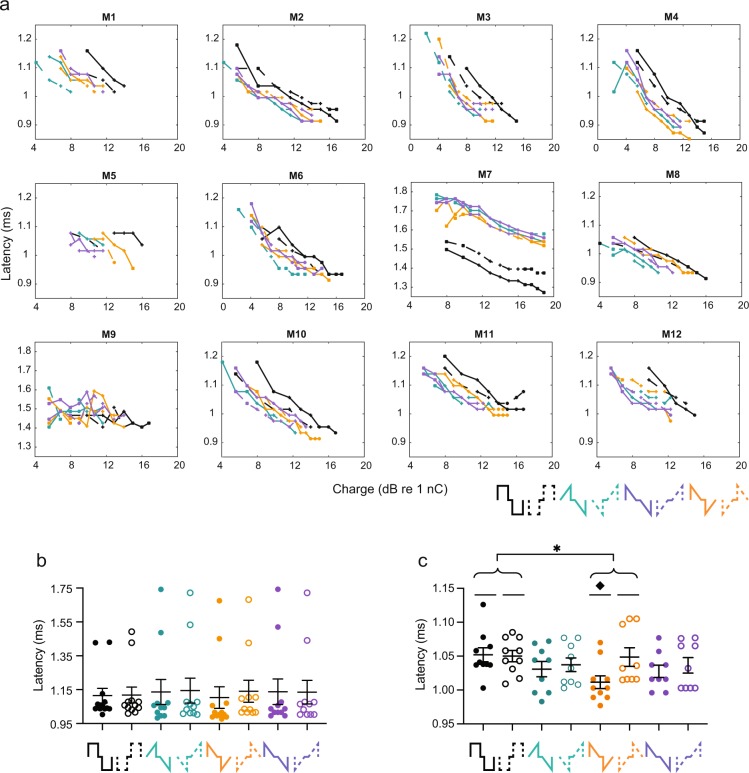
rampDOWN have shorter wave II latency than Rec. (**a**) Individual wave II latencies as a function of charge. Mouse (M)1–12. Pulse shapes are indicated by color and shown in the legend (bottom right). Solid line, anodic-first; dashed, cathodic-first. Note the longer latency with M7 and M9. (**b**) Wave II latency at three dB above threshold. (**c**) Wave II latency at three dB above threshold with M7 and M9 excluded. Pulse shapes are drawn on the x-axis. Each circle is data from one mouse. Closed circles, anodic-first pulses; open circles, cathodic-first pulses. Asterisk and diamond denote significant effect in post-hoc test of the factor pulse shape and the pulse shape*polarity interaction (Tukey HSD), respectively. *p ≤ 0.05; ^♦^p ≤ 0.05. n = 12. Error bars are SEM.

The latency of wave II depends on several factors, including numbers of synchronized fibers, excitation site along the auditory nerve fibers (central vs peripheral site), degree of temporal jitter, membrane properties, electrode-to-neuron distance, various stimuli pulse parameters (such as pulse shape, interphase gap, polarity)^25^ or a combination of these factors. For instance, anodic pulses produce shorter latencies compared to cathodic pulses in both human^25–28^ and animal^2,29^ physiological recordings, which is suggested to be due to excitation of the soma or central axon with anodal stimulation and of the peripheral processes with cathodic stimulation. To test if ramped and rectangular pulses evoke similar response latencies, we measured wave II latencies with increasing stimuli level (Fig. 3a). All mice showed slightly shorter latencies with Rec pulses compared to ramped shapes, except for mice M7 and M9, at measurable thresholds. To study suprathreshold latencies across animals, we examined the latency at three dB above threshold (Fig. 3b). Pulse shape (P = 0.7705), polarity (P = 0.3627), or shape*polarity (P = 0.0640) did not have a significant effect on wave II latency. The wave II latency of mice M7 and M9 for all eight waveforms were within 2 SD, but not within 1 SD, of the group mean. Nevertheless, after excluding these two animals from the statistical tests, we found that pulse shape did have a significant effect on the latency (P = 0.0220) (Fig. 3c). Post hoc analysis (Tukey HSD) also showed that rampDOWN had a significantly shorter wave II peak latency (mean latency: 1.029 ms) than did Rec (mean latency: 1.051 ms). However, the difference between these two latencies was 22 µs, which is close to the recording resolution of one sample (20 µs) and could therefore just be noise. The pulse shape*polarity interaction was also found to be significant (P = 0.0160). Post hoc analysis (Tukey HSD) showed that the latency of rampDOWN-anodic-first (mean latency: 1.012 ms) was on average 37 µs, corresponding to ∼2 samples (40 µs), shorter than those of Rec-anodic-first, Rec-cathodic-first and rampDOWN-cathodic-first (mean latency: 1.052 ms, 1.050 ms, and 1.046 ms, respectively).

There could be several possible explanations for the difference in latency. First, the delay between the onset of the first and second phases of the pulse shape is 35 µs which is close to the observed averaged latency difference with rampDOWN-A of 37 µs. One could therefore speculate that the neuronal membrane is excited by the first anodic phase in rampDOWN-A and by the second phase in Rec-A, Rec-C and rampDOWN-C. In addition, action potentials evoked by rampDOWN-A could be less influenced by the phenomenon of ‘anodal blocking’^30–32^. Here, the stimulus elicits an action potential near the terminal of the nerve fiber, but the stimulus itself prevents its propagation due to a strong hyperpolarization of the more distant and central part of the fiber. Second, the shorter latency could be caused by excitation of more central sites on the auditory nerve. Finally, shorter latency could imply less temporal jitter, and thus a stronger synchronized activity. However, these possible explanations relate to single fibers. As eABRs reflect the summed activity of many neurons, single- or multi-unit recordings and higher sampling frequencies are needed to get further insight into the mechanisms behind the potential latency differences with ramped pulse shapes. Thus, although our results show that pulse shapes give rise to significant different latencies, they should be considered with caution as eABR is evoked activity across many neurons, and because the differences we observed were in the order of 1–2 samples. The effects that pulse shape and polarity have on latency is a topic of future research.

### RampUP is more charge-efficient than Rec with longer phase duration

Previous studies have found that firing thresholds for single auditory fibers^22,33^, eABRs^34^, eCAPs^35,36^ and psychophysical detection level thresholds^37,38^ decrease with increasing pulse phase duration. If the neuronal membrane was a perfect integrator of charge, one would expect the threshold to decrease by 6 dB with a doubling of the phase duration (i.e. a halving of amplitude current level for doubling phase duration and corresponding to a constant amount of charge). However, the abovementioned studies and others have consistently shown that this effect is <6 dB, which suggests that the neuronal membrane is a ‘leaky’ integrator of charge. Sensitivity to pulse phase duration also reflects temporal integration of charge and is possibly related to other temporal measures, such as sensitivity to rapid envelope fluctuations or temporal fine structures^37^.

**Figure 4:**
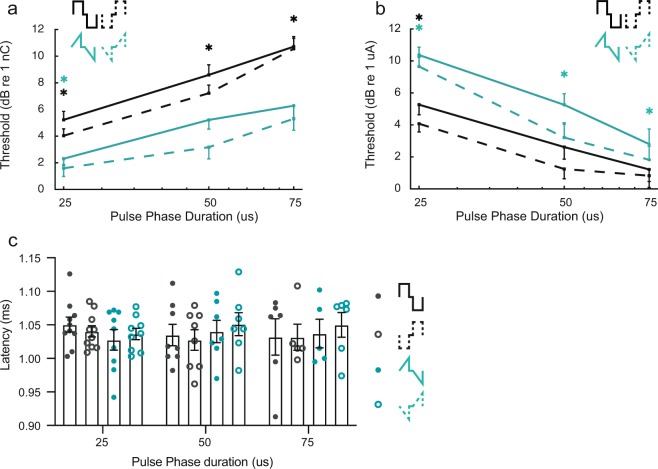
RampUP is more charge-efficient than Rec at longer phase durations. Thresholds of increasing phase durations (25 µs, 50 µs, 75 µs) with Rec (black) and rampUP (green) in terms of (**a**) amplitude current level and (**b**) charge injected. Solid line, anodic-first pulses; dashed, cathodic-first pulses. n = 12 mice for 25 µs/phase and 50 µs/phase, n = 9 mice for 75 µs/phase. Black and green asterisks denote significant effect in post hoc test of the pulse shape*polarity interaction (Tukey HSD) of Rec and rampUP, respectively. *p ≤ 0.05. Only one error bar is shown for simplicity. (**c**) Latency of wave II at three dB (re 1 nC) above threshold for ramp Rec (black) and rampUP (green). Each circle is data from one mouse. M7 and M9 are excluded. Closed circles, anodic-first pulses; open circles, cathodic-first pulses. n = 10 mice for 25 µs/phase, n = 7–8 mice for 50 µs/phase, n = 5–6 mice for 75 µs/phase.

To test if ramped pulses are also more charge-efficient at longer pulse durations and to explore how ramped pulses are integrated over time, we presented two additional phase durations of 50 µs and 75 µs and recorded eABRs for Rec and rampUP pulses. Thresholds were reported in dB re 1 nC and dB re 1 µA and plotted against phase duration on a log scale (Fig. 4a,b, respectively). With increasing pulse duration, lower amplitude current levels (Fig. 4b) but larger amounts of charge (Fig. 4a) were needed to reach threshold. Thus, the fact that the amount of charge is not constant (not a horizontal line) demonstrates there is a leaky integration of charge with both Rec and rampUP (Fig. 4b). A mixed model reported that all three factors - polarity (P = 0.0116), pulse shape (P < 0.0001), and phase duration (P < 0.0001) – had a significant effect on the threshold charge, as did phase duration*shape (P < 0.0001). Post hoc analysis showed lower threshold for cathodic-first than for anodic-first pulses (Student’s t-test) and for rampUP compared to Rec (Student’s t-test). This finding supports the previous results obtained with a phase duration of 25 µs (Fig. 2b). Furthermore, all three phase durations were different from each other (Tukey HSD), with the lowest threshold in charge for 25 µs, followed by 50 µs, and then 75 µs phase width stimuli, respectively. Regarding the phase duration*shape relation, all three Rec pulse durations were different from each other, with the lowest threshold in charge for Rec-25 µs, followed by Rec-50 µs, and then Rec-75 µs phase width stimuli, respectively. Meanwhile, 25 µs-rampUP produced a significant lower threshold in charge than did 50 µs-rampUP and 75 µs-rampUP stimulation did (Tukey HSD). Statistical analysis of the current threshold values showed that polarity (P = 0.0017), pulse shape (P < 0.0001) and phase duration (P < 0.0001) had similar significant effect on the phase duration, as did duration*polarity (P = 0.0004). Regarding the latter, all three durations of rampUP were significantly different, while 50 µs and 75 µs were significantly different from 25 µs Rec stimulation in terms of current (Tukey HSD).

As mentioned above, a perfect integration of charge would imply a slope of the strength-duration function of 0 dB re 1 nC/doubling (constant amount of charge). To test the integration of charge with rampUP and Rec pulses, we performed linear regression of the strength-duration functions to obtain the slopes (mean slope values: 3.45 dB re 1 nC/doubling for Rec-A (r^2^ = 0.99); 4.01 dB re 1 nC/doubling for Rec-C (r^2^ = 0.99); 2.55 dB re 1 nC/doubling for rampUP-A (r^2^ = 0.99); 2.26 dB re 1 nC/doubling for rampUP-C (r^2^ = 0.95); −2.56 dB re 1 µA/doubling for Rec-A (r^2^ = 0.99); −2.14 dB re 1 µA/doubling for Rec-C (r^2^ = 0.94); −3.47 dB re 1 µA/doubling for rampUP-A (r^2^ = 0.99), and −3.76 dB re 1 µA/doubling for rampUP-C (r^2^ = 0.98)). The dB re 1 nC/doubling slope values were positive for both Rec and rampUP pulses, but lower for rampUP. This means that rampUP pulses are also more charge-efficient than Rec pulses at longer phase durations, despite the fact that the ramp of rampUP is shallower at longer durations (lower peak current level). Overall, the results show that less charge is needed to compensate for membrane leakage over the phase duration of rampUP compared to Rec pulses.

Finally, if the cathodic polarity is more efficient than the anodic polarity, we would expect a shift in latency for anodic-first pulses with longer phase durations, but not for cathodic-first pulses. To test this, we analysed the latency of wave II as a function of pulse phase duration (Fig. 4c). A mixed model showed that pulse shape, phase duration and polarity had no significant effect on the latency of wave II, in contrast to the effect that polarity had in the threshold data reported above. One possible explanation could be that the eABR recording resolution of 20 µs/sample is too low to detect the expected latency shift of e.g. 50 µs when increasing the phase duration from 25 µs to 75 µs.

### The effect with ramped pulses is not due to an “extra” interphase gap but rather to the ramp itself

As mentioned previously, several studies have shown that increasing the interphase gap reduces behavioural and physiological thresholds^2,20–22^. One interpretation of the results with ramped pulses could therefore be that the effect is due to an “extra” interphase gap rather than sensivity to the ramp itself. Here, an “extra” interphase gap is understood as low level parts of the ramp that is below the threshold and ineffective in terms of neural excitation and thus presumably act as an “extra” interphase gap. Following this, a ramped pulse shape would be as efficient as a rectangular pulse shape with a longer interphase gap, shorter pulse duration and higher amplitude (with the same charge as a ramped pulse shape). This idea would fit with the shorter latencies observed with rampDOWN, and with the lower thresholds obtained with rampLONG (but rampUP) pulse shapes.

To test this hypothesis, we presented a biphasic pulse with a long interphase gap of 2.9 ms in the second group of mice (n = 6). In this way, the interaction between the two phases is minimized in a charge-balanced manner.

**Figure 5:**
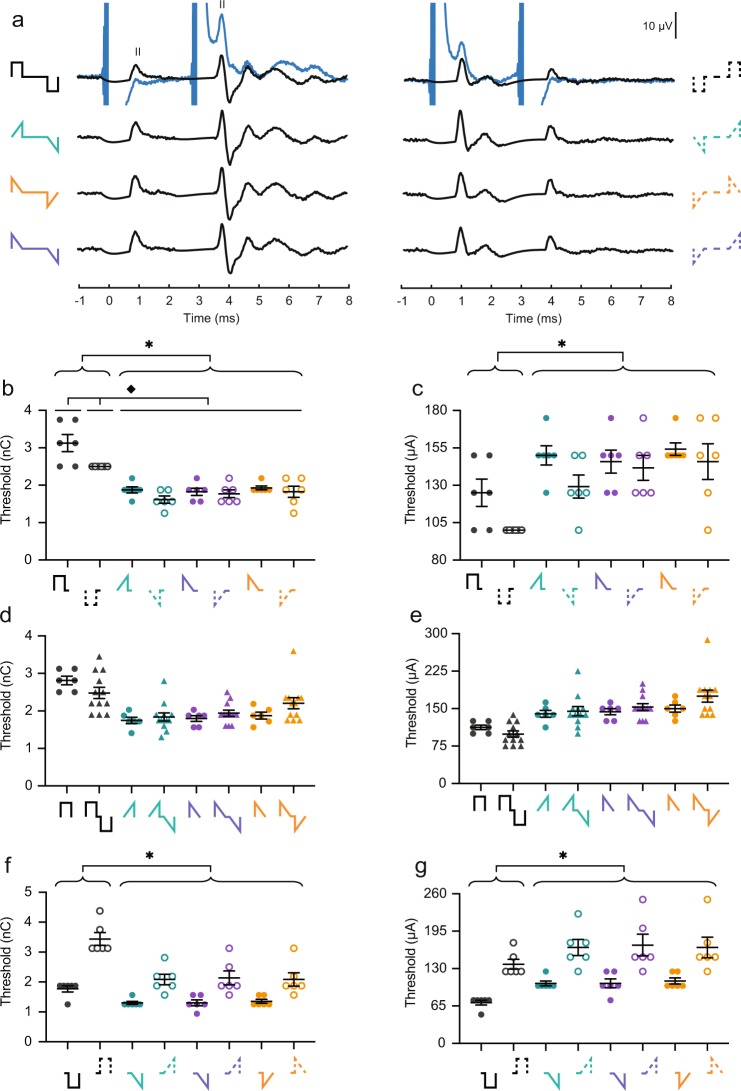
The effect with ramped pulses is not due to an “extra” interphase gap but rather to the ramp itself. (**a**) Example eABR traces to (left) Rec-A, rampUP-A, rampDOWN-A, rampLONG-A and (right) Rec-C, rampUP-C, rampDOWN-C, rampLONG-C with an interpulse gap of 2.9 ms. The amount charge injected was 3.6 nC for rectangular pulses and 2.5 nC for ramped pulses. The raw trace before removal of the exponential trend (blue line) is shown for Rec-A and Rec-C. Data from Mouse M15. (**b,c**) Thresholds of wave II evoked by the first, monophasic phase in (**b**) charge and (**c**) amplitude current level. Pulse shapes are shown below the x-axis. Each circle is data from one mouse. Closed circles, anodic-first pulses; open circles, cathodic-first pulses. (**d,e**) Thresholds of wave II evoked by the first, monophasic wave (circles) and by the biphasic pulse with an interpulse gap of 10 µs (triangle, replotted from Fig. 3b) in (**d**) charge and (**e**) amplitude current level. Both conditions were averaged across polarity. Only anodic-first shapes are shown below the x-axis for simplicity. (**f,g**) Thresholds of wave II evoked by the second, monophasic phase in (**f**) charge and (**g**) amplitude current level. Asterisk and diamond denote significant effect in post-hoc test of the factor pulse shape and the pulse shape*polarity interaction (Tukey HSD), respectively. *p ≤ 0.05; ^♦^p ≤ 0.05. n = 6. Error bars are SEM.

An eABR was triggered at the first phase of both anodic - and cathodic-first rectangular shapes (Fig. 5a). This fits with previous findings where both the anodic and cathodic phase in a rectangular pulse can be excitatory, as demonstrated in model predictions^23^, in animal auditory nerve recordings with monophasic pulses^2^ and in human eABR recordings with pseudomonophasic pulses^25^. In contrast, eCAP studies in human CI listeners have only found evidence of neural activation by the anodic phase in an asymmetric pulse^26,27,39^. An eABR could also be evoked by the first phase of both polarities of the ramped pulse shapes (Fig. 5a), which demonstrates that both anodic and cathodic ramped pulses can be excitatory.

Next, we analysed the threshold of wave II evoked by the first phase (Fig. 5b). A mixed model reported that pulse shape (n = 6, P < 0.0001) and polarity (P = 0.0442) were significant factors on the threshold in terms of charge. The pulse shape*polarity interaction was also significant (P = 0.0296). Post hoc comparison showed that the threshold was lower for all three ramped pulses compared to Rec (P < 0.05, Tukey HSD) and lower for the cathodic phase compared the anodic phase (Student t-test). The statistical analysis also showed that the threshold was higher for Rec-A than for Rec-C, and that Rec-A and Rec-C produced higher threshold than all ramped pulses (Tukey HSD). The threshold of the first, monophasic phase (averaged across polarity) was similar to those obtained using a biphasic pulse with interphase gap of 10 µs (averaged across polarity) for the ramped pulses, but slightly higher for the monophasic Rec shape compared to the biphasic Rec shape (Fig. 5d). This result is surprising as most animal and human studies suggest that monophasic and pseudomonophasic pulses are more effective than biphasic ones^22,25,27,33,40^. One explanation could be the overall good health of the peripheral processes in the acutely deafened mice used in this study.

We also analysed wave II evoked by the second, monophasic phase (Fig. 5f). A mixed model showed a highly significant effect of both pulse shape (n = 6, P < 0.0001), polarity (P = 0.0030) and pulse shape*polarity (P = 0.0008) on the threshold. Similar to the results for the first phase, posthoc analysis showed that Rec had a higher threshold than all three ramped pulses (P < 0.05, Tukey HSD) and that there was no difference between the ramped pulse shapes. Post hoc analysis also showed that anodic-first pulses (with a cathodic second phase) produced lower thresholds than cathodic-first pulses (with an anodic second phase) (Student t-test), which again demonstrates higher sensitvitity to the cathodic phase.

In terms of amplitude current level (Fig. 5c,g), pulse shape was a significant factor on the threshold evoked by both the first and second phase (both p < 0.0001). Post hoc comparison showed that the threshold of Rec was significantly lower than that of all three ramped pulses for both phases (Tukey HSD), similar to the results in terms of charge (Fig. 5b,f). Polarity had only a signifianct effect on the threshold response evoked by the second phase polarity (P = 0.0049), with lowest threshold for anodic-first pulses (with a cathodic second phase) (Student t-test). This suggests that a monophasic cathodic pulse preceded by a monophasic anodic pulse evokes wave II at lower levels than a cathodic pulse alone in terms of current level amplitude (Fig. 5g), and thus that an initial anodic phase may facilitate the response following the cathodic phase despite a long interphase gap of 2.9 ms.

**Figure 6:**
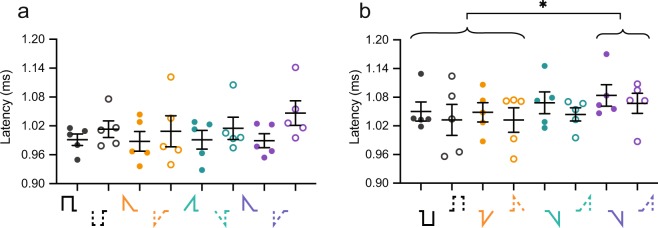
Monophasic ramped and rectangular pulse shapes evoke similar wave II latencies. (**a**) Latency of wave II evoked by the first, monophasic phase at three dB above threshold. (**b**) Latency of wave II evoked by the second, monophasic phase at three dB above threshold. Asterisk denote significant effect in post-hoc test of the factor pulse shape (Tukey HSD). *p ≤ 0.05. n = 6. Error bars are SEM.

Finally, we also measured the latency of wave II at three dB above threshold evoked by both the first (Fig. 6a) and second phase (Fig. 6b). A mixed model showed that neither pulse shape nor polarity had a significant effect on the latency on the first phase, in line with the healthy peripheral process of the animals, but that pulse shape had a significant effect on the latency on the second phase (P = 0.0063). Further post hoc comparison showed that Rec and rampDOWN were significantly different from rampLONG (Tukey HSD). Nonetheless, rampLONG and rampUP have the same second phase (increasing ramp), so the fact that rampUP is not different from Rec and rampDOWN suggest that the observed latency difference between Rec and rampDOWN and rampLONG could simply be due to the low sample number and the effect of one outlier.

Thus, less charge, but higher current amplitude, is needed to evoke wave II with ramped pulses with a long interpulse gap compared to rectangular pulses. These results support the interpretation that the reduced threshold with ramped pulses is due to the ramp itself rather than an “extra” interphase gap. In contrast to the results with biphasic pulses, we found no effect of the shape of the ramped, monophasic pulse on the threshold of wave II evoked at the first phase and second phase.

## Discussion

Our study presents the first physiological data on CI-stimulation with ramped pulse shapes. We found that less charge is needed with ramped pulse shapes than with rectangular pulse shapes to evoke an eABR response of similar amplitude. Here, rampUP, a pulse shape with a rising ramp over both phases, was most efficient in terms of charge, i.e. less charge was needed to produce a response (Fig. 2b,d). Cathodic-first pulses had lower thresholds than anodic-first pulses (Fig. 2b,c), which was also demonstrated in traces with a 2.9 ms interpulse gap (Fig. 5). Interestingly, the latter stimulation paradigm also showed that both anodic and cathodic phases could evoke an eABR response, a finding that is in accordance with the literature, and that the benefit with ramped pulses is due the ramp itself rather than an ‘extra’ interpulse gap (Fig. 5b,c,f,g). Finally, we showed that rampUP was also more charge-efficient at longer pulse durations compared to Rec shapes (Fig. 4c). These findings support the hypothesis that ramped pulse shapes can be beneficial in terms of lower charge consumption, which has the potential to reduce battery usage in human CIs.

Ballestro *et al*. demonstrated a proof-of-concept by using biophysically-inspired pulse shapes for CI stimulation^4^. In patch-clamp recordings, they found that the firing probability of cultured SGNs increased with a gradually rising slope of ramped pulse shape^4^. But comparing this result with ours is not straightforward because there are several methodological differences. First, Ballestro *et al*. recorded firing probability in cultured SGN where an electrode provides a direct input to a single neuron. In contrast, eABR is a gross measure of neural activity, reflecting synchronous activity of those fibers most sensitive to low-level stimuli. Thus, there are several processing steps that have to be taken to go from what is being presented at the intracochlear electrode to what the SGN actually receives. Those steps include impedance in the electrode, fluid and bone, electrode position relative to the peripheral processes, site of excitation, extent of myelination, spread of excitation, temporal and spatial integration, etc. Second, in contrast to our study, Ballestro *et al*. did not control for the amount of charge injected nor did they test for declining ramped pulses to enable a fair comparison with rectangular pulses. Thus, the observed higher firing probability they observed with steeper slopes could simply be due to more injected charge. Third, Ballestro *et al*. used a ramped pulse with a pedestal or so-called foot amplitude (Fig. 2a in Ballestro *et al*.^4^). Their hypothesis was that the foot amplitude of a given amplitude drives SGNs to subthreshold and that the steepness of the ramp then is used to control the evoked firing rate. In contrast, we used a purely ramped stimulus in which the stimulus phases ramped either up from 0 µA to x µA or ramped down from x µA to 0 µA. Fourth, Ballestro *et al*. used a monophasic pulse with long phase duration and slow presentation rate (100 µs anodic pulse presented at 0.33 pps), while in our study we used charge-balanced biphasic pulses with short phase durations and a faster presentation rate (25 µs/phase presented at 23.3 pps). That said, clinical presentation rates are higher (e.g. 900 pps) than what is used for eABRs, and the effects of ramped pulses therefore need to be further evaluated with clinically relevant rates.

Nonetheless, a higher firing probability with steeper slope in cultured SGNs could imply a lower eABR threshold, as observed in this study with rampUP and rampLONG pulses. Also, the fact that rampUP had lower thresholds than rampDOWN supports the hypothesis that a rising slope is important for efficient stimulation. For example, Yip *et al*. used a genetic-algorithm of mammalian myelinated auditory nerve fibers to generate the most energy efficient waveforms^5^. An exponentially-decaying cathodic phase and an approximately rectangular anodic phase yield energy savings of 20–30% compared to a biphasic rectangular waveform below 50 μs phase width, and also yield energy savings of ~40% for pulse widths between 125 μs and 180 μs^5^. This waveform therefore likely takes advantages of the fact that animals are most sensitive to the cathodic phase^2,41^ and that the action potential therefore is triggered during this phase. In contrast, the rectangular anodic shape does not likely contribute to the firing, but rather ensures charge-balance. Yip *et al*. also performed a simple loudness task with biphasic exponential waveform (decaying exponential cathodic, growing exponential anodic), which is comparable to our cathodic-first rampLONG result. They found a 26% charge reduction compared to using rectangular pulses at mid-loudness level. In support of this, we found a 24% charge reduction in eABR threshold with rampLONG-C compared to rectangular pulses (Fig. 2b). In general, our fidings are in line with the literature. We found a lower threshold with ramped pulses than with rectangular pulses, and we found that a rising ramp is beneficial. That said, it is important to stress that the ramped current slope used in this study was not perfect, but stepwise, due to hardware limitations.

Most studies with human CI users and with animals have demonstrated a good correlation between eABR and psychophysical thresholds^42–45^, although there is a large variability across and within subjects, and with different methodological approaches. We would therefore also expect lower psychophysical threshold with ramped pulses in mice, as observed in our eABR data.

In this study, the steeper growth of wave II with ramped pulses that we observed suggests that maximum stimulation (most comfortable level) can be obtained with less charge. Yet, it would only be beneficial if the number of discriminable loudness level steps are, at least, maintained. In our eABR recordings, the upper limit of electric stimulation was in most mice determined by the presence of a facial nerve response and the numbers of intensity steps is unknown. The potential of a reduced dynamic range and thus reduced power consumption needs to be further investigated with psychophysical data.

We also found that rampUP pulses produced a significantly steeper slope than rampDOWN. This indicates that the direction (rising or declining) of the stimulus ramp matters. As mentioned, a steeper growth function implies a higher recruitment rate of neurons with increasing intensity. Various mechanisms have been found to contribute to slope steepness. First, activation of auditory nerve fibers is known to depend on the electrode configuration, as demonstrated by greater eABR growth with monopolar stimulation than with bipolar stimulation^19,34,42^. One suggested explanation for this is that there are different sites of excitation along the SGN. A more confined bipolar stimulation fields is proposed to activate sites on the peripheral processes, while monopolar stimulation activates more central sites on the SGN^46^. Thus, activation of more densely placed SGNs at the modulus might account for the steeper growth function observed with monopolar stimulation as stimulus level increases^34^. The site-of-excitation is also influenced by level. In particular, bipolar activation is proposed to switch from a peripheral to a more central site on auditory nerve fibers. This switch is supported by eABR latency data that show that latencies are shorter with monopolar than with bipolar stimulation^44^.

Second, Shepherd *et al*. found steeper eABR I/O functions in cats when stimulating with an electrode close to spiral ganglion neurons than with an electrode close to dendritic processes^19^. These data were supported by Frijns *et al*. in their modelling of electrical stimulation in guinea pigs, which showed faster recruitment of fibers with somatic than with dendritic excitation. Their model also predicted a lower threshold and higher spatial selectivity with electrodes in the dendritic position^30,47^. Based on this, one could speculate that ramped pulses excite sites on the auditory nerve fiber distinct from rectangular pulses to produce steeper growth function. Interestingly, immunostainings show that Kv.1.1and Kv1.2 subunits of the ramp-sensitive KLT channels localize to SGN somata^7^, but whether there is a relation between ramped stimulation, KLT localization and site of excitation has yet to be determined. Indeed, it would be interesting to explore how and at which site ramped and rectangular pulses excite the auditory nerve fibers, using latency data from single- or multiunit neuronal recordings. Also, if ramped stimuli activate KLT channels, which promotes a sharply timed action potential^48^, then we would expect there to be less temporal jitter in the firing of single neurons close to the stimulated electrode.

Third, a faster recruitment of more neurons (and thus a steeper slope) could also be explained by a larger current spread, which goes against the hypothesis that ramped pulses create a more focused stimulation. Spread of excitation with ramped pulses is not addressed in the study, but is an appealing topic of future research.

It is known that both the anodic and cathodic phase in a rectangular pulse can elicit neural responses^2,25,49^. In human CI listeners, studies have shown that at supra-thresholds, the anodic phase is more efficient than the cathodic phase^25,27,50^. In contrast, animals show greater sensitivity to the cathodic phase^2,49^. The reason for this discrepancy is yet to be clarified but computational models suggest two possible explanations^30,51^. First, that little polarity effects are observed in healthy SGNs (with the cathodic phase being the more efficient) while stronger differences are present in degenerated and demyelinated peripheral processes (with the anodic phase being the more efficient)^51^. Second, that at high levels of cathodic stimulation, the propagation of the spike is blocked by a strong hyperpolarization at more central sites of the fiber, which eventually leads to higher levels needed with cathodic pulses than anodic pulses. The phenomenon has been termed “cathodal blocking”^30^. In the present study, we found that cathodic-first biphasic pulses were 1.10 times more efficient than wtith anodic-first pulses at threshold. The result supports previous animal, not human, studies and is likely related to the relatively healthy peripheral processes in our acutely deafned mice. This is an important point in terms of clinical translation.

**Figure 7:**
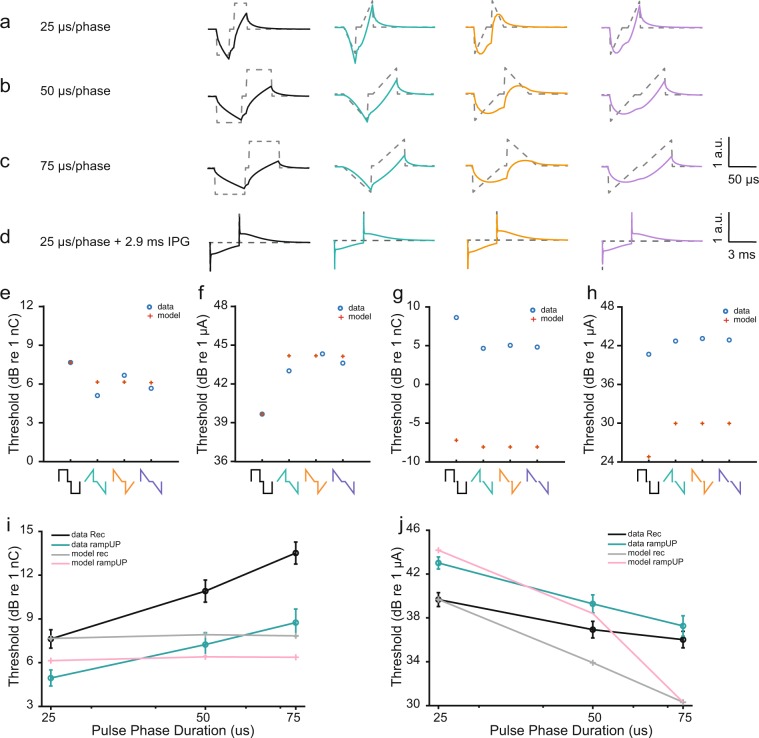
Comparison of measured wave II thresholds and predicted thresholds from the lowpass filter model. (**a–d**) Input waveform (dashed, grey) and the filtered signal (solid, color) plotted on top for phase durations of (**a**) 25 µs (**b**) 50 µs and (**c**) 75 µs and (**d**) an interpulse gap of 2.9 ms with Rec (black), rampUP (green) rampDOWN (orange) and rampLONG (purple). Note the input level has an arbitrary unit. Only cathodic-first polarity is shown. (**e,f**) Threshold in charge (**e**) and current (**f**) from the mouse data (averaged across polarity and across mice) of wave II (blue circle) and model predictions (red cross) with 25 µs/phase and an interpulse gap of 10 µs for all four waveforms. n = 12. (**g,h**) Threshold in current (**g**) and charge (**h**) from mouse data (averaged across polarity and of 1^st^ and 2^nd^ phase, across mice) of wave II (blue circle) and model predictions (red cross) with 25 µs/phase and an interpulse gap of 2.9 ms for all four waveforms. n = 6. (**i,j**) Threshold in current (**j**) and charge (**j**) from the mouse data (averaged across polarity and across mice) of wave II (black; Rec, green; rampUP) and model predictions (grey; Rec, pink; rampUP) as a function of phase durations. n = 12 for 25 µs/phase and 50 µs/phase, n = 9 for 75 µs/phase. Error bars are SEM.

Carlyon *et al*. presented a simple low pass model to describe reduced behavioral thresholds with increasing interphase gaps in CI users and in animals^21^. We therefore hypothesized that such a model could predict the lower thresholds we observed with ramped pulses and could be used as a simple tool to understand how the waveform being integrated by SGNs actually looks. Details of our model can be found in Methods and in Carlyon *et al*.^21^. In short, it passes pulse shapes through a simple lowpass filter. The predicted threshold then corresponds to a fixed RMS level at the filter output. Carlyon *et al*. used a so-called animal-filter to model behavioral thresholds in cats for different frequencies of sinusoidal electrical stimulation^52^. We implemented this model by using the animal filter as an attempt to explain the eABR threshold that we observed, without taking the slope-sensitivity into account (thus, without modelling KLT channel dynamics; see e.g. references^10,23^ for detailed auditory nerve models). It is important to note that our model does not take polarity effects into consideration. Interestingly, the filtered output of rampUP and Rec looked similar for the three phase durations (Fig. 7a–c, black and green), while the filtered output of rampDOWN looked more rectangular than ramped (Fig. 7a–c, orange). Our model predicted lower charge thresholds for all three ramped pulses compared to Rec (Fig. 7e, red cross), which was in accordance with the animal data (Fig. 7e, blue circle). In contrast, our model did not capture the observed higher threshold we observed with rampDOWN. Instead, rampUP and rampDOWN thresholds that we predicted were the same because of identical power spectra. With respect to phase duration, our model predicted that the amount of charge would be constant as a function of phase duration (Fig. 7i, grey and pink line) and thus did not capture the ‘leaky’ integration of charge as found in the mouse (Fig. 7i, black and green line). Nonetheless, less charge was needed with rampUP compared to Rec for longer phase durations (Fig. 7i, grey and pink line). In contrast to the decrease in threshold with increasing interphase gap found in other studies^21,22^, our model predicted much smaller threshold values (difference of ∼8–11 dB) for pulses with a long interphase gap (Fig. 7d) compared to the animal data (Fig. 7g,h). The results show the limitations of the model. First, that the model is calibrated to a reference point in the animal data (see Methods). Second, that the cut-off frequencies in the filter model, which are based on behavioural thresholds in cats, might not fit physiological thresholds. This suggests that the time course over which electrical charge is recovered is different for physiological and behavioural thresholds. Third, that the model is not adapted to mice. For instance, Carlyon *et al*. modelled that the behavioural threshold in cats would drop with increasing interphase gap over a substantially shorter time course than in human^21^. One could therefore speculate that there also would be species differences relevant for integration of charge between cats and mice, which is not implemented in the model. The model could, however, capture the difference in threshold between rectangular and ramped pulses observed in the mouse in terms of current level amplitude (Fig. 7h) but less in terms of charge (Fig. 7g).

Overall, this simple lowpass model could show differences in thresholds between ramped and rectangular pulses with a short interpulse gap without taking the slope-sensitivity into account.

The present study was performed in mice. To put the results into a clinical perspective, we will highlight four major differences between mice and humans. First, mice use a higher frequency range of hearing than humans do. Stimulating the most apical electrode target relatively mid-high frequencies, around 30 kHz^53^. However, it seems reasonable to expect similar benefits of ramped pulses also at lower frequencies. Second, other key differences between mice and humans are the length of the peripheral processes and the degree of myelination of the soma. Indeed, the health status of spiral ganglion neurons is thought to be the major reason why human CI users show lower threshold for anodic than cathodic pulses, as discussed above. Third, the scala tympani is smaller in mice than it is in humans, which makes it possible to position the electrode closer to the neuronal processes and thereby stimulate the auditory nerve more efficiently in the mouse, as found in a study in cats^19^. This might produce a larger spread of excitation from one electrode in mice and possibly underestimate the potential beneficial effect that ramped pulses might have on spatial selectivity. Finally, the small head size of mice is likely to contribute to a more sensitive eABR measure compared to those obtained in humans. The differences between ramped and rectangular pulse shapes observed in mice may therefore be larger in humans. Nonetheless, the data presented in our study show that it is possible to use ramped pulse shapes for CI stimulation but more work is needed to investigate their clinical relevance.

Taken together, our results show that rampUP pulses at the level of the brainstem have a higher charge-efficiency. This provides useful insights into the general understanding of the electrode-neuron interface. It also has the potential to lower battery usage in CI’s, and it may open new perspectives when designing other efficient neural implants in the future.

## Materials and Methods

### Subject

All animal procedures were approved by the Veterinary Office of the Canton of Basel, Switzerland and complied with guidelines established by the Basel University, Switzerland. C57BL/6 adult mice, aged 8–12 weeks, were used in this study. The animals were housed under a 12 hr/12 hr light/dark cycle and had access to food and water *ad libitum*. The left ear was used as the experimental ear. Details of the surgical approach and electrode array are provided in Navntoft *et al*.^54^.

### Surgery

Mice were anesthetized with ketamine (80 mg/kg) and xylazine (16 mg/kg) (volume injected at 10 μl/g body weight, i.p.). Supplemental anesthesia was given with a lower dose of ketamine (45 mg/kg, injected at 10 μl/g body weight, i.p.). Local analgesic (0.1 mg/ml bupivacaine and 0.4 mg/ml lidocaine, 0.1 ml injected s.c.) was administered along the intended incision line to minimize any surgical discomfort. Body temperature was maintained at 36.6 °C for the duration of the experiment by using a heating pad (FHC, ME, USA).

A post-auricular incision was made and the sternocleidomastoid muscle was retracted to reveal the tympanic bulla periosteum. The bulla was then opened and further extended dorsally to visualize the round window niche. Animals were deafened to eliminate electrophonic responses. 0.2 ml 0.5% weight/volume neomycin (neomycin trisulfate salt, N1876-25G, Lot#WXBB7516V, Sigma, dissolved in saline and adjusted to pH = 7.5) was slowly perfused through the round window. Next, a small piece of spongostan soaked in neomycin was placed within the round window niche and an acoustic ABR was recorded 45 minutes afterwards.

A 4-channel electrode array (platinum bands of Ø0.2 mm, spaced at 0.4 mm interval, Bionics Institute, Melbourne, Australia) was inserted into the scala tympani until the 4^th^ platinum ring was located just inside the round window. This gave an insertion depth of ~2 mm, corresponding to an intracochlear position at ~30 kHz^53^. The lead wire was then coiled inside the bulla and the incision was closed. A silver wire ground ball was placed in a subcutaneous pocket in the neck.

### Stimuli

Stimuli were generated with a custom-made software program developed by Oticon Medical (Oticon Medical, Vallauris, France) and delivered to the implanted electrode array by an Animal Stimulator Platform also provided by Oticon Medical (Oticon Medical, Vallauris, France). Trigger pulses for each stimulus were generated by the Animal Stimulator Platform and recorded by a digital signal processor from Tucker Davis Technologies (RZ6, TDT, FL, USA). The impedance of each electrode was measured before and after the recording session to ensure that all electrodes were functioning. The stimulation current was for all animals always below the compliance limit of respective electrode contact.

Four pulse shapes and two polarities giving eight waveforms were tested (Fig. 1a). The rectangular pulse shape (Rec) has, as its name indicates, a rectangular shape with a flat phase amplitude. The three ramped shapes are defined according to their slope, specifically the rate at which the injected current increases or decreases linearly over time. In rampUP, for both the first and the second phase, the slope ramped from zero at the phase onset to a specified amplitude current level at the phase offset. In rampDOWN, for both the first and the second phases, the slope ramped from a specified amplitude current level at the phase onset to zero at the phase offset. In rampLONG, for the first phase, the slope ramped from a specified amplitude current level at the phase onset to zero at the phase offset. For the second phase, the slope ramped from zero at the phase onset to a specified amplitude current level at the phase offset. Thus, with rampUP, rampDOWN and rampLONG we respectively tested the effect that a rising, declining and longest possible slope could have. Also, rampDOWN and rampLONG have the same shape for the first phase, but opposite in the second phase, and rampUP and rampLONG have the same second phase, but opposite first phase. Both anodic- and cathodic-first polarities were tested.

Finally, it is important to stress that the ramped current slope was not smooth but approximated in current step size of 25 μA due to a hardware limitation with our Animal Stimulator Platform. The temporal resolution of the Animal Stimulator Platform was 3–4 μs. Charge per phase for rectangular pulses was calculated as pulse width times current level amplitude, and for ramped pulses it was calculated as pulse width times current level amplitude divided by two.

## Auditory Brainstem Response (ABR)

### Acoustic-evoked ABR (aABR)

aABR were used to determine hearing threshold before and after deafening with neomycin. ABR recordings were performed in soundproof and electrically-shielded booth under anesthesia as detailed above. The contralateral (right) ear was blocked with acoustic foam (Otoform® Ak, Dreve Otoplastik GmbH) in order to isolate the ABR response from the ipsilateral (left) ear. Click trains (512 reps: 0.1 ms monophasic clicks; 0–90 dB peak equivalent Sound Pressure Level, SPL, in 10 dB steps; 21 Hz presentation rate) were generated in BioSig software from Tucker Davis Technologies (TDT, FL, US) and by a digital signal processor (RZ6, TDT, FL, USA), and presented via a calibrated MF1 speaker (TDT, FL, USA) positioned at 10 cm away from the animal’s left ear. ABR were recorded by using stainless steel electrodes positioned at the vertex, hind leg, and behind the pinna of the ipsilateral ear (active, ground and reference, respectively). All electrode impedances were kept below 3 kΩ. Responses were amplified through a Medusa TDT pre-amplifier (RA4PA, TDT, FL, US) with a gain of 20, and then recorded with a sampling frequency at 24′414 Hz and with resolution of 16 bit/sample. Responses were filtered offline by a bandpass Butterworth (4 order) at 100–3′000 Hz using custom made software in Matlab (MathWorks, MA, USA). The ABR threshold was determined visually as the lowest dB level with a recognizable ABR wave response.

### Electrically-evoked ABR (eABR)

eABRs were used to measure the response to various pulse shapes. The recording procedure for eABRs was the same as for aABRs (described above), except for the use of a PZ5 multimodal NeuroDigitizer amplifier (TDT, FL, US), which has a larger input range (500 mV) to avoid clipping by the stimulus artefact. The sampling rate was 48′828 Hz.

eABR stimuli were charge-balanced biphasic pulses. In the first group of animals (n = 12), we used Rectangular, rampUP, rampDOWN, rampLONG (Fig. 1a) with a phase duration of 25 μs and an interphase gap of 10 μs. We also used Rectangular and rampUP with a phase duration of either 50 μs or 75 μs, and an interphase gap of 10 μs. In the second group of animals (n = 6), we used Rectangular, rampUP, rampDOWN, rampLONG with a phase duration of 25 μs and an interphase gap of 2.9 ms. To obtain a higher sampling resolution of the eABR response, we used an up-sampling technique. The sample unit with 48′828 Hz is 20.48 μs. To up-sample the data, four recordings were made at each current level with the pulse delayed by 0 μs, 5 μs, 10 μs, and 15 μs, respectively. The delay was then corrected and the four recordings were averaged offline to obtain an approximated sampling rate of 48′828 Hz times four. Four hundred repetitions were presented at each stimulus level and delivered at a rate of 23.3 pulses per second (pps) in monopolar mode on the most apical electrode. Both anodic- and cathodic-first pulses were tested, with anodic-first having an anodic phase followed by a cathodic phase and with cathodic-first having a cathodic phase followed by an anodic phase. The conditions were presented in randomized order. Stimulation artefacts are shown in Supplementary Fig. 4. At the end of the data collection period, animals were euthanized with a lethal injection of pentobarbital (300 mg/ml, 0.1 ml injected i.p.).

### Data analysis

Both eABR and aABRs were processed with a custom-made software program in Matlab (Mathworks, MA, USA). Averaged aABRs from the BioSig Software program were bandpass filtered (4th order Butterworth) with cut-off frequencies set to 100 Hz and 3000 Hz. The stimulus artefact in eABR was removed by using linear interpolation before the signal was bandpass filtered in a way similar to that for aABR (Supplementary Fig. 5). Epochs were then extracted, baseline corrected and averaged. In order to cancel the stimulation artefact from traces with an interpulse gap of 2.9 ms, which depends on pulse shape, an exponential function was fitted from 0.3 until 2.7 ms (first phase) and from 3.2 until 11 ms (second phase) after the onset of the stimuli. The traces were hereafter linearly interpolated and bandpass filtered in a way similar to that for eABR with an interpulse gap of 10 μs. Time zero was defined as the onset of the click stimulus in aABR and as the onset of the stimulus artefact in eABR. aABR responses were quantified by the amplitude and by the latency of wave II, which is generated in the cochlear nuclei. eABR wave II-IV, which are generated in the cochlear nuclei, superior olivary complex and lateral leminiscus, respectively, were quantified. Amplitude was defined as the voltage difference between the positive peak and the following trough. Latency was defined as timing of the peak. Threshold was determined as the lowest charge level needed to evoke a wave amplitude above 0.1 μV. To ensure that no remaining stimulus artefact was present at wave II, an alternating polarity artefact reduction was performed. The data for each pulse shape was averaged over both polarities to cancel out the polarity-symmetric stimulus artefact. We verified that the thresholds and growth function of the averaged data were between the thresholds and growth function for the single polarities, respectively (Supplementary Fig. 6). That said, we cannot exclude a potential remaining fully-decayed stimulus artefact at higher stimulation levels. However, this should not affect the threshold value and the slope analysis. Growth functions were fitted with either a linear or a ‘broken stick’ function, using the Curve Fitting Toolbox from Matlab, based on visual inspection. The minimum and maximum values for ‘broken stick’ fitting parameters were as follows: [0.1 50] μV/nC for the slope of line 1, [−2 0] μV/nC for the slope of line 2, [−10 2] μV for crossing with the y-axis for line 1, and [2 20] nC for the x-value of the knee point, respectively.

Thus, the slope of line 2 was forced to be 0 or negative to get the saturation point of the growth function.

eABR threshold and slope values were averaged across each polarity to get one threshold and slope value for each of the four pulse shapes.

### Filter model

Our filter model is based on the model developed by Carlyon *et al*.^21^ with minor adjustments. In brief, our model assumes that the electric waveform is passed through a lowpass filter and that the threshold corresponds to a fixed RMS level of the filter output. We used an biquadratic IIR filter with a flat frequency response from 0 Hz to 110 Hz, then with an attenuation of 6 dB per octave between 110 Hz and 4000 Hz, and after 4000 Hz an attenuation of 3 dB per octave. Each of the four waveforms (Rec, rampUP, rampDOWN, rampLONG) that we used were filtered by using Matlab filter design tools. The filter output was then multiplied by a series of Hanning windows, each with a duration of 10 ms. The window was moved 1/10 of its length and the root mean square (RMS) output of each window calculated. The windows spanned one period of the stimulus. That period was the same as the one used in the eABR recordings (giving a presentation rate of 23.3 Hz). Finally, thresholds were assumed to be inversely proportional to the maximum of the RMS output on any window. The output was reported in dB re an arbitrary unit. To match the model output to the animal data, the threshold of the model (RMS target value) was defined such that a modelled Rec shape pulse with 25 μs/phase and an interpulse gap of 10 μs produced similar threshold as the same pulse shape (averaged across polarity) measured in the mouse.

### Statistical analysis

To analyse and compare the overall effects of amplitudes and latencies of responses, factor analyses were performed by using a mixed model with random effects in the statistical software program called JMP (version 14.1, SAS Institute, Cary, NC). Normality and homogeneity were checked by visual inspecting plots of residuals against fitted values. The pulse shape, polarity and pulse duration were fixed factors, while mouse was set as a random factor. A paired t-test was used to test the aABR threshold before and after neomycin. Correlations between aABR and eABR thresholds and growth function slopes were tested for with a Pearson correlation and a two-tailed p-value. The level of significance was α = 0.05 in all statistical tests. ﻿All data are reported as mean ± standard error of the means (SEM).

## Supplementary information

Supplementary Information.

## Data Availability

Data are available upon reasonable request.

## References

[1] Friesen LM, Shannon RV, Baskent D, Wang X (2002). Speech recognition in noise as a function of the number of spectral channels: Comparison of acoustic hearing and cochlear implants. J. Acoust. Soc. Am..

[2] Miller CA, Abbas PJ, Robinson BK, Rubinstein JT, Matsuoka AJ (1999). Electrically evoked single-fiber action potentials from cat: Responses to monopolar, monophasic stimulation. Hear. Res..

[3] Rubinstein JT, Miller CA, Mino H, Abbas PJ (2001). Analysis of monophasic and biphasic electrical stimulation of nerve. IEEE Trans. Biomed. Eng..

[4] Ballestero J (2015). Reducing current spread by use of a novel Pulse shape for electrical stimulation of the auditory nerve. Trends Hear..

[5] Yip M, Bowers P, Noel V, Chandrakasan A, Stankovic KM (2017). Energy-efficient waveform for electrical stimulation of the cochlear nerve. Sci. Rep..

[6] Recugnat, M., Undurraga, J. & McAlpine, D. Abstract: Modelling electrically evoked compound action potential of single neuron responses to predict polarity and pulse shape effects on spiral ganglion neurons under electro-stimulation. In *Conference on Implantable Auditory Prostheses* (2019).

[7] Smith KE, Browne L, Selwood DL, McAlpine D, Jagger DJ (2015). Phosphoinositide modulation of heteromeric Kv1 channels adjusts output of spiral ganglion neurons from hearing mice. J. Neurosci..

[8] Mo ZL, Adamson CL, Davis RL (2002). Dendrotoxin-sensitive K+ currents contribute to accomodation in murine spiral ganglion neurons. J. Physiol..

[9] Negm MH, Bruce IC (2014). The effects of HCN and KLT ion channels on adaptation and refractoriness in a stochastic auditory nerve model. IEEE Trans. Biomed. Eng..

[10] Boulet J, Bruce IC (2017). Predictions of the vontribution of HCN half-maximal activation potential heterogeneity to variability in intrinsic adaptation of spiral ganglion neurons. J. Assoc. Res. Otolaryngol..

[11] Ferragamo M, Oertel D (2002). Octopus cells of the mammalian ventral cochlear nucleus sense the rate of depolarization. J. Neurophysiol..

[12] Joshi, S., Marozeau, J., Epp, B. & Carney, L. Poster: Low-threshold potassium channels and their effect on polarity sensitivity of the electrically stimulated auditory nerve. *CIAP Meet*. *2017*

[13] Dorman MF, Loizou PC (1997). Speech intelligibility as a function of the number of channels of stimulation for normal-hearing listeners and patients with cochlear implants. Am. J. Otol..

[14] Fu QJ, Nogaki G (2005). Noise susceptibility of cochlear implant users: The role of spectral resolution and smearing. J. Assoc. Res. Otolaryngol..

[15] Schvartz-Leyzac KC, Zwolan TA, Pfingst BE (2017). Effects of electrode deactivation on speech recognition in multichannel cochlear implant recipients. Cochlear Implants Int..

[16] Croghan NBH, Duran SI, Smith ZM (2017). Re-examining the relationship between number of cochlear implant channels and maximal speech intelligibility. J. Acoust. Soc. Am..

[17] Lotfi Navaii M, Sadjedi H, Jalali M (2013). Waveform efficiency analysis of auditory nerve fiber stimulation for cochlear implants. Australas. Phys. Eng. Sci. Med..

[18] Claussen AD (2019). A mouse model of cochlear implantation with chronic electric stimulation. PLoS One.

[19] Shepherd RK, Hatsushika S, Clark GM (1993). Electrical stimulation of the auditory nerve: The effect of electrode position on neural excitation. Hear. Res..

[20] Miller AL, Smith DW, Pfingst BE (1999). Across-species comparisons of psychophysical detection thresholds for electrical stimulation of the cochlea: II. Strength-duration functions for single, biphasic pulses. Hear. Res..

[21] Carlyon RP (2005). Effect of inter-phase gap on the sensitivity of cochlear implant users to electrical stimulation. Hear. Res..

[22] Shepherd RK, Javel E (1999). Electrical stimulation of the auditory nerve: II. Effect of stimulus waveshape on single fibre response properties. Hear. Res..

[23] Joshi SN, Dau T, Epp B (2017). A model of electrically stimulated auditory nerve fiber responses with peripheral and central sites of spike generation. J. Assoc. Res. Otolaryngol..

[24] Bahmer A, Baumann U (2016). The underlying mechanism of preventing facial nerve stimulation by triphasic pulse stimulation in cochlear implant users assessed with objective measure. Otol. Neurotol..

[25] Undurraga JA, Carlyon RP, Wouters J, Van Wieringen A (2013). The polarity sensitivity of the electrically stimulated human auditory nerve measured at the level of the brainstem. J. Assoc. Res. Otolaryngol..

[26] Undurraga JA, Carlyon RP, Macherey O, Wouters J, van Wieringen A (2012). Spread of excitation varies for different electrical pulse shapes and stimulation modes in cochlear implants. Hear. Res..

[27] Macherey O, Carlyon RP, Van Wieringen A, Deeks JM, Wouters J (2008). Higher sensitivity of human auditory nerve fibers to positive electrical currents. J. Assoc. Res. Otolaryngol..

[28] Spitzer ER, Hughes ML (2017). Effect of stimulus polarity on physiological spread of excitation in cochlear implants. J. Am. Acad. Audiol..

[29] Miller CA (2004). Intracochlear and extracochlear ECAPs suggest antidromic action potentials. Hear. Res..

[30] Frijns JHM, De Snoo SL, Kate JH (1996). Spatial selectivity in a rotationally symmetric model of the electrically stimulated cochlea. Hear. Res..

[31] Ranck JB (1975). Which elements are excited in electric stimulation of mammalian central nervous system: a review. Brain Res..

[32] Macherey O, Carlyon RP, Chatron J, Roman S (2017). Effect of pulse polarity on thresholds and on non-monotonic loudness growth in cochlear implant users. J. Assoc. Res. Otolaryngol..

[33] Shepherd RK, Hardie NA, Baxi JH (2001). Electrical stimulation of the auditory nerve: Single neuron strength-duration functions in deafened animals. Ann. Biomed. Eng..

[34] Miller CA, Woodruff KE, Pfingst BE (1995). Functional responses from guinea pigs with cochlear implants. I. Electrophysiological and psychophysical measures. Hear. Res..

[35] Ramekers D (2014). Auditory-nerve responses to varied inter-phase gap and phase duration of the electric pulse stimulus as predictors for neuronal degeneration. J. Assoc. Res. Otolaryngol..

[36] Prado-Guitierrez P, Fewster LM, Heasman JM, McKay CM, Shepherd RK (2006). Effect of interphase gap and pulse duration on electrically evoked potentials is correlated with auditory nerve survival. Hear. Res..

[37] Chatterjee M, Kulkarni AM (2014). Sensitivity to pulse phase duration in cochlear implant listeners: Effects of stimulation mode. J. Acoust. Soc. Am..

[38] Shannon RV (1985). Threshold and loudness functions for pulsatile stimulation of cochlear implants. Hear. Res..

[39] Undurraga JA, van Wieringen A, Carlyon RP, Macherey O, Wouters J (2010). Polarity effects on neural responses of the electrically stimulated auditory nerve at different cochlear sites. Hear. Res..

[40] Wieringen AV (2005). Effects of waveform shape on human sensitivity to electrical stimulation of the inner ear. Hear. Res..

[41] Macherey O, Van Wieringen A, Carlyon RP, Deeks JM, Wouters J (2006). Asymmetric pulses in cochlear implants: Effects of pulse shape, polarity, and rate. J. Assoc. Res. Otolaryngol..

[42] Abbas PJ, Brown CJ (1991). Electrically evoked auditory brainstem response: Growth of response with current level. Hear. Res..

[43] King J, Shehu I, Roland JT, Svirsky MA, Froemke RC (2016). A physiological and behavioral system for hearing restoration with cochlear implants. J. Neurophysiol..

[44] Miller CA, Faulkner MJ, Pfingst BE (1995). Functional responses from guinea pigs with cochlear implants II. Changes in electrophysiological and psychophysical measures over time. Hear. Res..

[45] Smith DW, Watt S, Konrad KEM, Olszyk VB (2005). Behavioral auditory thresholds for sinusoidal electrical stimuli in the cat. J. Acoust. Soc. Am..

[46] van den Honert C, Stypulkowski PH (1987). Single fiber mapping of spatial excitation patterns in the electrically stimulated auditory nerve. Hear. Res..

[47] Frijns JHM, de Snoo SL, Schoonhoven R (1995). Potential distributions and neural excitation patterns in a rotationally symmetric model of the electrically stimulated cochlea. Hear. Res..

[48] McGinley MJ, Oertel D (2006). Rate thresholds determine the precision of temporal integration in principal cells of the ventral cochlear nucleus. Hear. Res..

[49] Klop WMC, Hartlooper A, Briare JJ, Frijns JHM (2004). A new method for dealing with the stimulus artefact in electrically evoked compound action potential measurements. Acta Otolaryngol..

[50] Carlyon RP, Deeks JM, Macherey O (2013). Polarity effects on place pitch and loudness for three cochlear-implant designs and at different cochlear sites. J. Acoust. Soc. Am..

[51] Rattay F, Lutter P, Felix H (2001). A model of the electrically excited human cochlear neuron. I. Contribution of neural substructures to the generation and propagation of spikes. Hear. Res..

[52] Miller AL, Smith DW, Pfingst BE (1999). Across-species comparisons of psychophysical detection thresholds for electrical stimulation of the cochlea: I. Sinusoidal stimuli. Hear. Res..

[53] Müller M, Von Hünerbein K, Hoidis S, Smolders JWT (2005). A physiological place-frequency map of the cochlea in the CBA/J mouse. Hear. Res..

[54] Navntoft CA, Marozeau J, Barkat TR (2019). Cochlear implant surgery and electrically-evoked auditory brainstem response recordings in C57BL/6 mice. JoVe.

