# Supplementary material for: Ramped pulse shapes are more efficient for cochlear implant stimulation in an animal model
Source: Sci Rep. 2020 Feb 24;10:3288. doi: 10.1038/s41598-020-60181-5 (PMC7039949; doi:10.1038/s41598-020-60181-5)
Supplement: Supplementary file 1 — Supplementary Information. [file 41598_2020_60181_MOESM1_ESM.pdf]

Ramped pulse shapes are more efficient for cochlear implant stimulation in an animal model

Charlotte Amalie Navntoft[1,2], Jeremy Marozeau[1], Tania Rinaldi Barkat[2,*]

[1]Hearing Systems Group, Department of Health Technology, Technical University of Denmark, Kgs. Lyngby, Denmark

[2]Brain and Sound Lab, Department of Biomedicine, Basel University, Basel, Switzerland

[*]Corresponding author: tania.barkat@unibas.ch

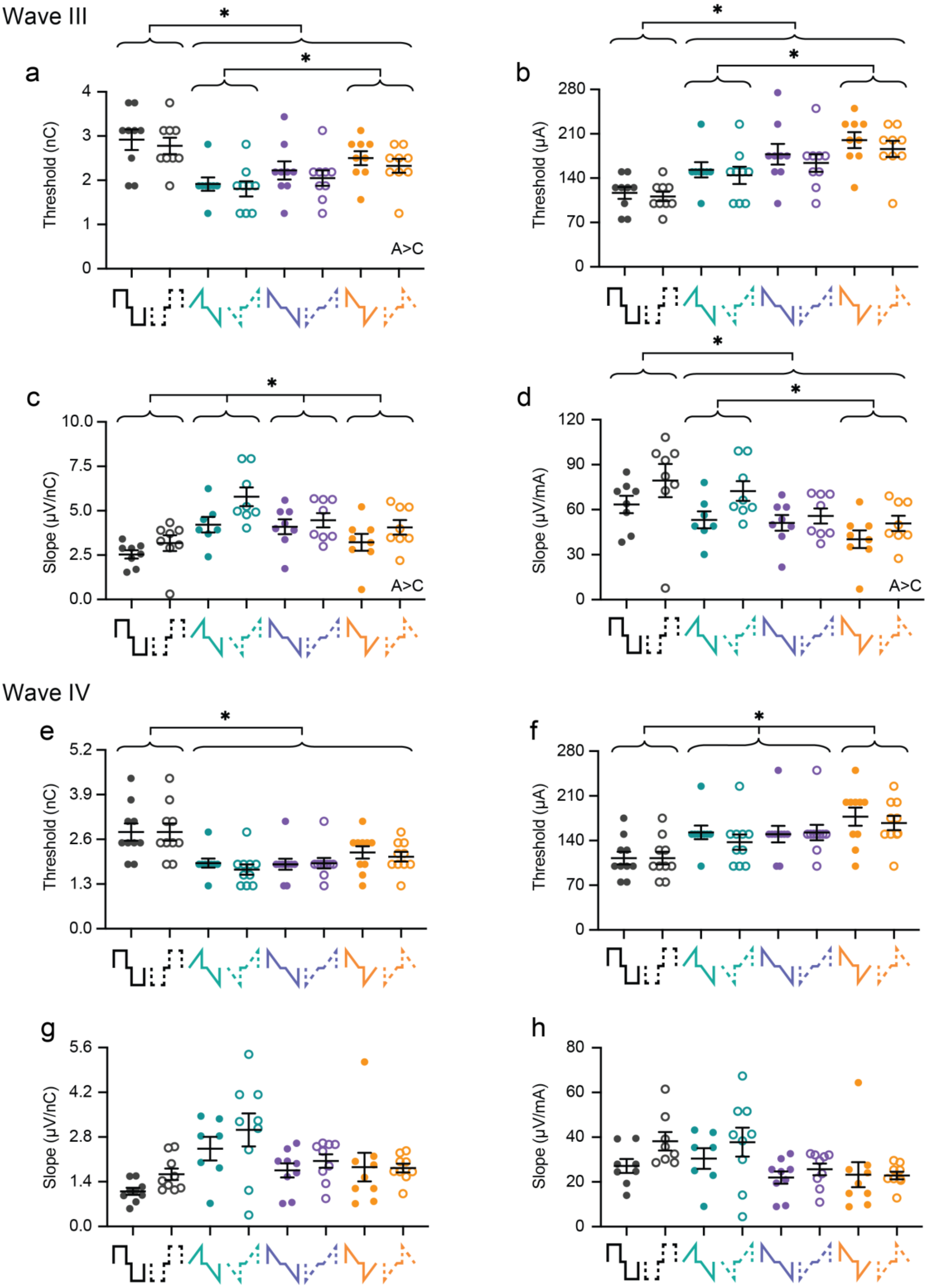

Supplementary Figure 1 eABR wave III and IV. (**a,b**) Thresholds of wave III in (a) charge and (b) amplitude current level. Pulse shapes are shown below the x-axis. Each circle is data from one mouse. Closed circles, anodic-first pulses; open circles, cathodic-first pulses. (**c,b**) Slopes of the growth function of wave III (c) in μV/nC and (d) μV/mA. (**e,f**) Thresholds of wave IV in (e) charge and (f) in amplitude current level. (**g,h**) Slopes of the growth function of wave IV in (g) μV/nC and (h) μV/mA. Asterisks denote significant effect of the factor pulse shape in a post-hoc test. *, p≤0.05. A>C denote a significant effect of the factor polarity in a post-hoc test. n=12. Error bars are SEM.

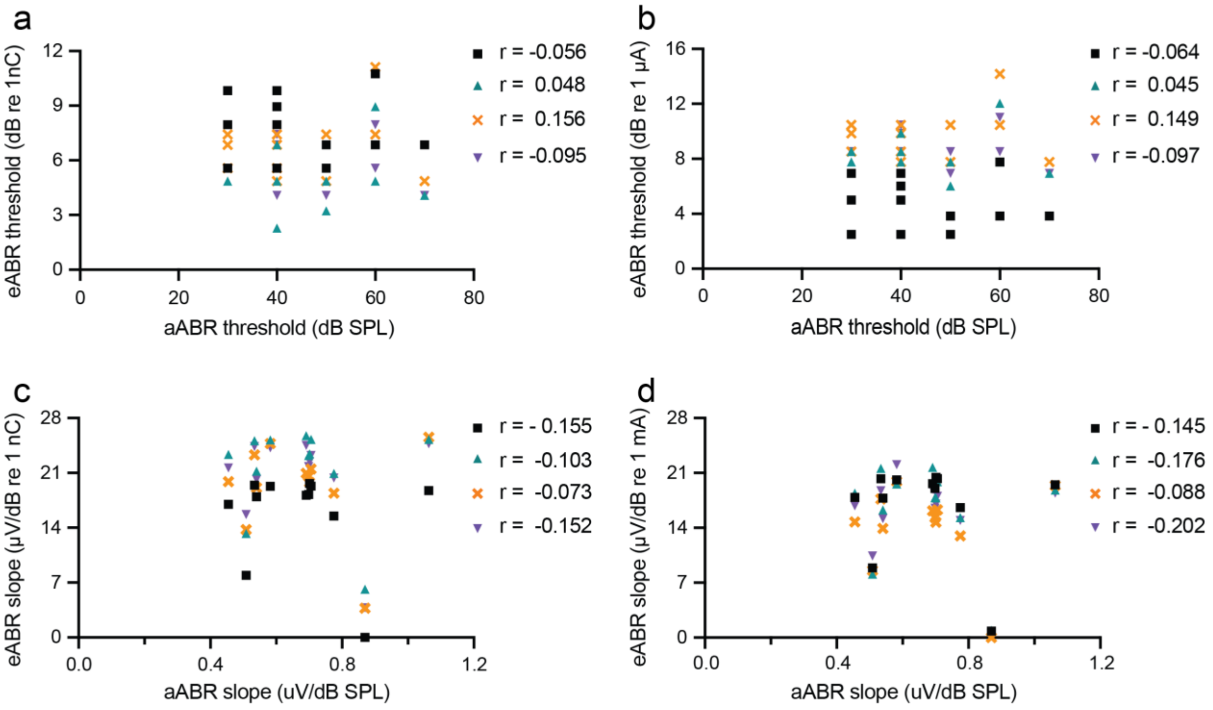

Supplementary Figure 2 eABR threshold and growth function slopes do not correlate with aABR. Correlation between aABR and eABR wave II thresholds in (**a**) charge and (**b**) amplitude current level. Correlation between aABR and eABR wave II growth function slopes in (**c**) charge and (**d**) amplitude current level. Each symbol is data from one mouse with eABR values averaged across polarity to obtain one value for each of the four pulse shapes. Black square; Rec, green upward triangle; rampUP, orange cross; rampDOWN, purple downward triangle; rampLONG. n=12. Error bars are SEM. Note the different threshold and slope units for eABR and aABR. Pearson correlations coefficients are shown on the right in each graph.

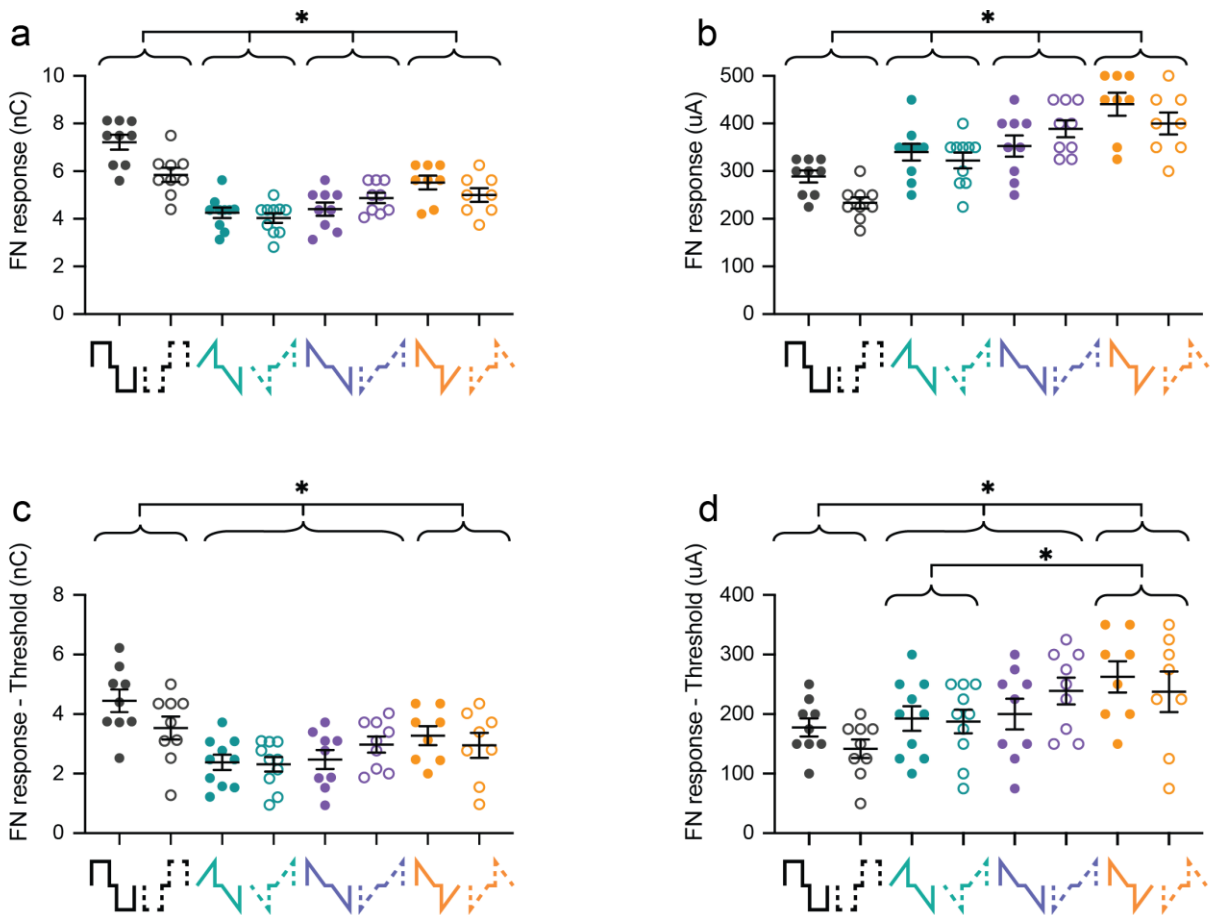

Supplementary Figure 3 Facial nerve responses as a function of pulse shape (25 μs/phase, 10 μs interpulse gap). (**a**) Thresholds of the facial nerve response in charge. Pulse shapes are shown below the x-axis. Each circle is data from one mouse. Closed circles, anodic-first pulses; open circles, cathodic-first pulses. Cathodic-first was significantly lower than anodic-first pulses (p≤0.05, not shown) (**b**) Thresholds of the facial nerve response in amplitude current level. (**c**) Dynamic range in charge defined as the facial nerve response minus the threshold of wave II. (**d**) Dynamic range in curent defined as the facial nerve response minus the threshold of wave II. Asterisks denote significant effect of the factor pulse shape in post-hoc test. *, p≤0.05. n=12. Error bars are SEM.

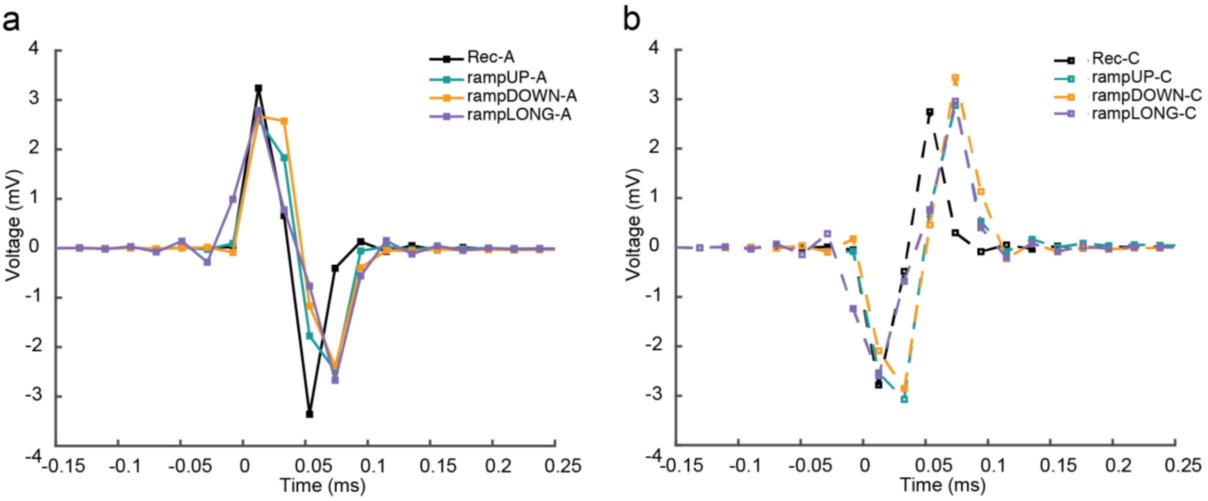

Supplementary Figure 4 Stimulation artefact from the eight pulse shapes used (25 μs/phase, 10μs interphase gap) at one current level (250 μA). The recordings were performed in a euthanized animal to avoid any neural responses. Note that the decaying stimulus artefact, which is removed using an averaged interpolation interval of 555 µs, is not visible here due to a larger scaling of the y-axis. (**a**) Anodic-first pulse shapes (solid line). (**b**) Cathodic-first pulse shapes (dashed line). Data from one mouse.

a

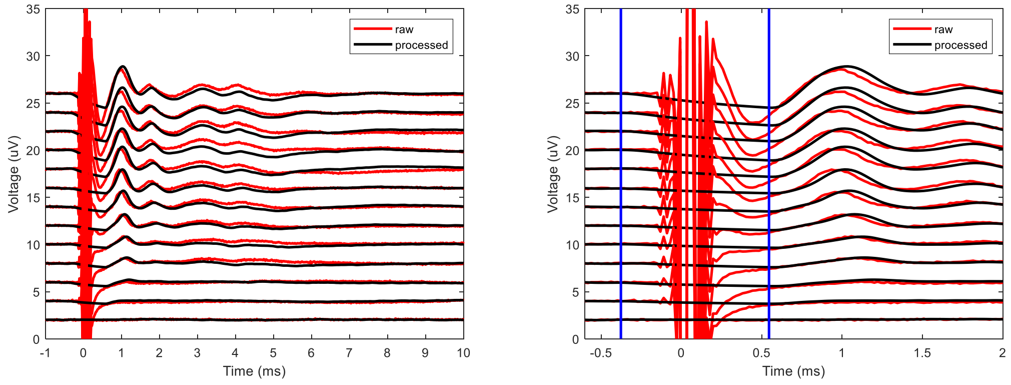

b

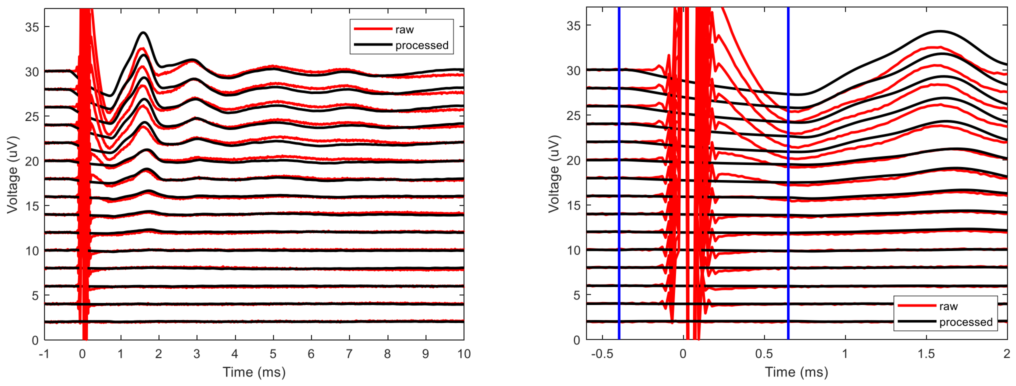

Supplementary Figure 5 Example of raw and processed eABR traces with 25 µs/phase and an interphase gap of 10 µs. (**a**) Rec-A presented at a current level of 0 µA and 75-350 µA in steps of 25 µA. Mouse 11. (**b**) rampUP-C presented at a current level of 0 µA and 75-600 µA in steps of either 25 µA, 50 µA or 100 µA. Raw traces (red), Processed traces after artefact removal by interpolation and filtering (black), start and end of interpolation (blue horizontal lines). The figures demonstrate the stimulation artifact decays before the occurrence of wave II at around 1 ms.

The mean, min and max time values of the end of the interpolation was 0.555 ms, 0.505 ms and 0.674 ms, respectively.

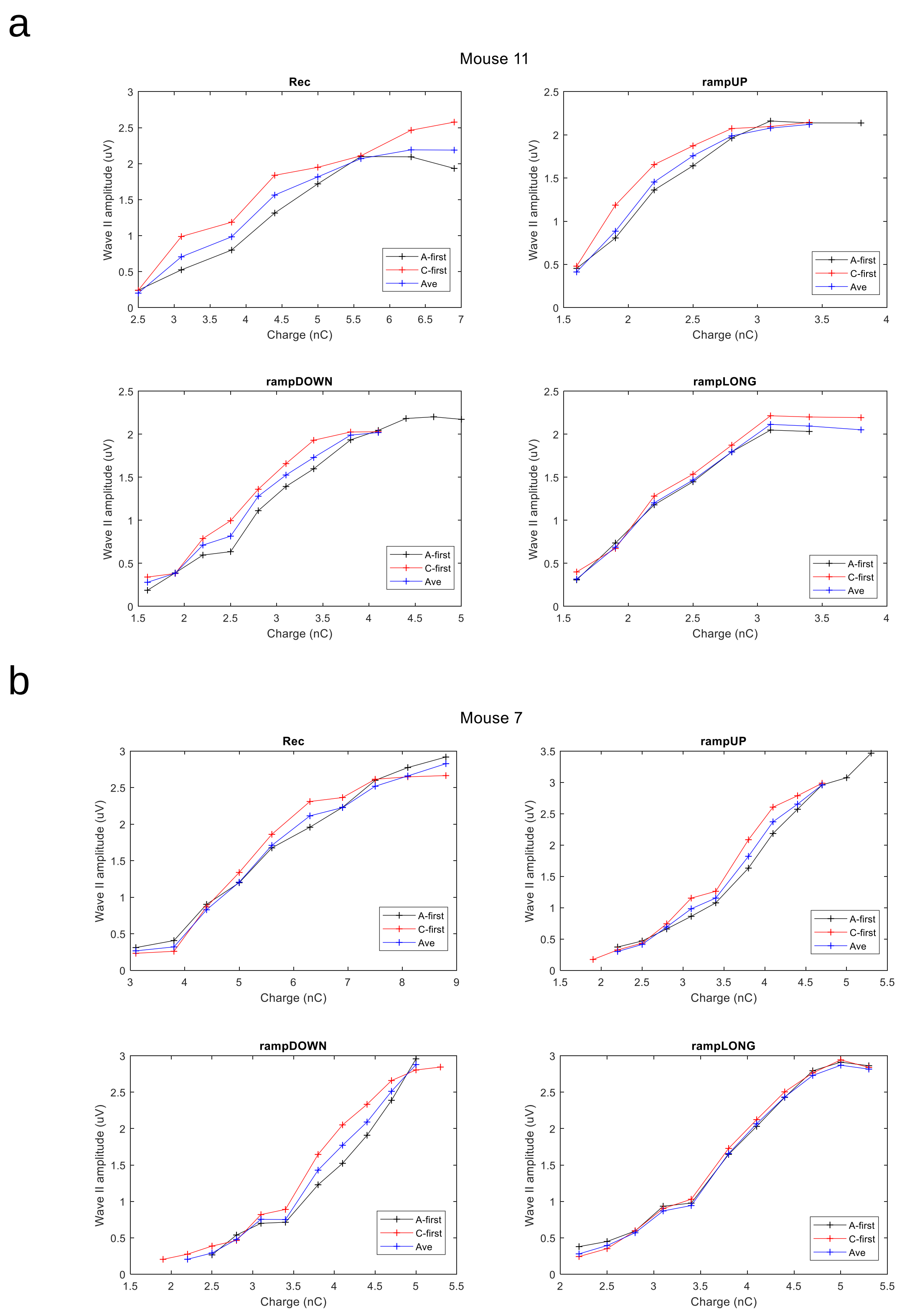

Supplementary Figure 6 Example of growth function of wave II from raw eABR traces. (**a**) Mouse 11. (**b**) Mouse 7. Anodic-first (black), cathodic-first (red) and averaged across polarity (blue) for all four pulse shapes used, Rec, rampUP, rampDOWN, rampLONG. The figures demonstrate that the averaged trace is between the traces from the two polarities suggesting that there is no polarity bias in the stimulation artefact.
